# 95. What To Do With Prolonged Bacteremia in *Staphylococcus aureus* Osteoarticular Infection? A Retrospective Analysis at Two Children's Hospitals

**DOI:** 10.1093/ofid/ofad500.011

**Published:** 2023-11-27

**Authors:** Jonathon C McNeil, Marritta Joseph, Jesus G Vallejo, Kristina G Hulten, Sheldon L Kaplan, Mary G Boyle, Stephanie A Fritz

**Affiliations:** Baylor College of Medicine, Houston, Texas; Baylor College of Medicine, Houston, Texas; Baylor College of Medicine, Houston, Texas; Baylor College of Medicine, Houston, Texas; Baylor College of Medicine, Houston, Texas; Washington University School of Medicine, St. Louis, Missouri; Washington University School of Medicine, St. Louis, Missouri

## Abstract

**Background:**

*Staphylococcus aureus* is the most frequent cause of osteoarticular infection (OAI, including hematogenous osteomyelitis and septic arthritis) in children and is commonly associated with bacteremia. Current guidelines suggest that persistent bacteremia may warrant more aggressive treatment, however, data supporting this are limited. We retrospectively evaluated the management and outcomes of children with *S. aureus* OAI with respect to bacteremia duration.

**Methods:**

Cases were identified through prospective *S. aureus* surveillance studies at Texas Children’s and St. Louis Children’s Hospitals. Children < 18 years old with OAI secondary to *S. aureus* from 2011-2021 were eligible. Prolonged bacteremia was defined *a priori* as the upper quartile of duration. Orthopedic complications included pathologic fractures, avascular necrosis, angular deformity and chronic osteomyelitis.

**Results:**

Overall, 532 cases were identified of which 343 (64.5%) had ≥1 positive blood culture; 61 developed orthopedic complications (11.4%, **Figure 1**). The median duration of bacteremia in the cohort was 1 day (IQR: 1-3 days). Patients with prolonged (≥ 3 days) and non-prolonged bacteremia were similar in terms of demographics. Children with prolonged bacteremia had a longer duration of fever in hospital (median 6 vs. 3 days, p< 0.001), more often had MRSA, pyomyositis and venous thromboses and were less often transitioned to oral therapy (**Figure 2**). Duration of bacteremia also influenced discharge antibiotic choice (**Figure 3**). Within the subset of patients with prolonged bacteremia, those who were transitioned to oral therapy had similar outcomes to those treated with prolonged intravenous antibiotics; such patients received a median of 11.5 days (IQR: 6-23) of IV therapy prior to discharge (median 7 days [IQR: 4-20] after last positive blood culture).

**Figure:**
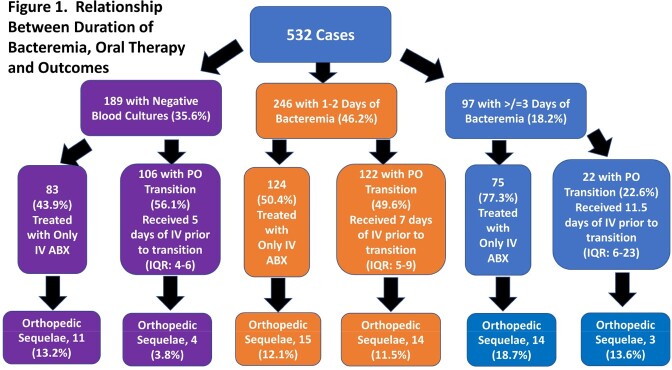

**Figure:**
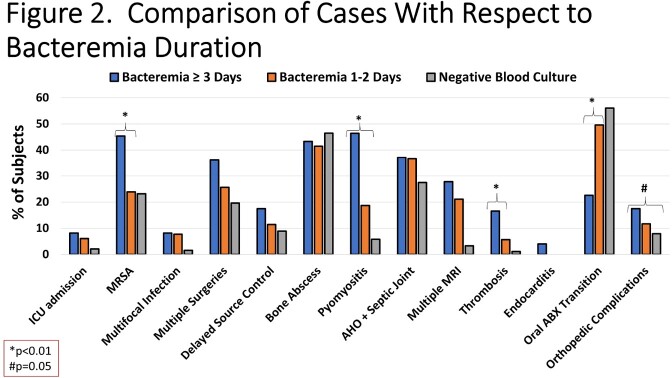

**Figure:**
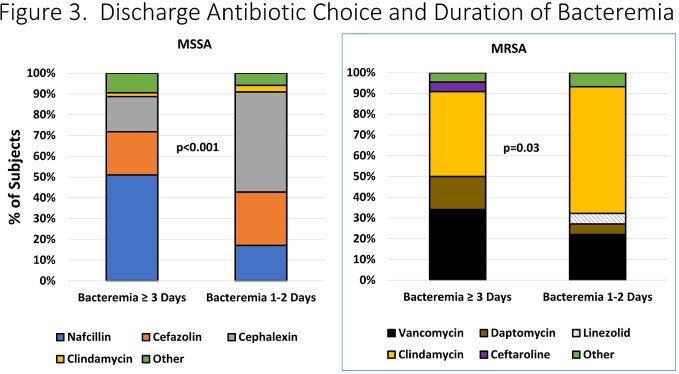

**Conclusion:**

Prolonged bacteremia in the setting of *S. aureus* OAI greatly impacts management and should prompt evaluation for other foci of infection. However, our data show that a subset of children with *S. aureus* OAI and prolonged bacteremia can be safely transitioned to oral antibiotics after a period of IV therapy. Additional work is needed to delineate optimal management of OAI with prolonged bacteremia.

**Disclosures:**

**Jonathon C. McNeil, MD**, Allergan: Grant/Research Support|Nabriva Therapeutics: Grant/Research Support **Kristina G. Hulten, PhD**, Pfizer: Grant/Research Support **Sheldon L. Kaplan, MD**, MeMed: Grant/Research Support|Pfizer: Grant/Research Support|Pfizer: Honoraria

